# Glutamate Metabotropic Receptors-Linked Postsynaptic Density Proteins: An Emergent Hub for Antipsychotics’ Regulation of Synaptic Plasticity and Metaplasticity

**DOI:** 10.3390/biom16020324

**Published:** 2026-02-19

**Authors:** Annarita Barone, Licia Vellucci, Anita Nasti, Benedetta Mazza, Federica Iannotta, Felice Iasevoli, Andrea de Bartolomeis

**Affiliations:** 1 Section of Psychiatry, Laboratory of Molecular and Translational Psychiatry, Unit of Treatment-Resistant Psychiatric Disorders, Department of Neuroscience, Reproductive Sciences and Dentistry, University of Naples “Federico II”, Via Pansini 5, 80131 Naples, Italy; annarita.barone@unina.it (A.B.); licia.vellucci@unina.it (L.V.); felice.iasevoli@unina.it (F.I.); 2 Department of Translational Medical Sciences, University of Naples “Federico II”, Via S. Pansini 5, 80131 Naples, Italy

**Keywords:** treatment resistant schizophrenia, metabotropic glutamate receptors, postsynaptic density, synaptic plasticity, antipsychotics, clozapine

## Abstract

Glutamate metabotropic receptors (mGluRs) and their molecular partners at the postsynaptic density (PSD) represent a highly dynamic molecular hub that integrates multiple neurotransmitter signals and regulates synaptic plasticity and metaplasticity, which are putatively involved in the pathophysiology of psychiatric illnesses, including schizophrenia. Group I mGluRs (mGluR1 and mGluR5) interact with PSD adaptor and scaffolding proteins, such as Homer, Shank, Norbin, and PICK1, as well as intracellular downstream effectors, creating a molecular network that resembles a Lego-like structure, where modular protein interactions fine-tune glutamatergic transmission. Evidence from preclinical research indicates that dysregulation of mGluR expression and function, along with disrupted PSD protein expression, may contribute to the pathophysiology of schizophrenia by altering glutamatergic neurotransmission and synaptic stability. Antipsychotic mechanisms of action may involve, at least in part, the modulation of mGluR activity mediated through PSD proteins. Notably, novel agents that enhance spinogenesis by acting at the level of PSD proteins, such as SPG302, may open promising avenues for therapeutics aimed at restoring synaptic integrity. While Group I mGluRs dominate postsynaptic regulation, Group II (mGluR2/3) and III (mGluR4/6/7/8) receptors -primarily presynaptic- inhibit neurotransmitter release and plasticity, offering complementary therapeutic avenues. Emerging strategies, such as allosteric modulators of mGluRs, aim to rebalance synaptic signaling in treatment-resistant schizophrenia. This review synthesizes how PSD proteins and mGluRs interact in schizophrenia, exploring their potential as druggable targets for novel therapies.

## 1. Introduction

Schizophrenia affects approximately 24 million individuals worldwide, according to the most recent estimates reported by the World Health Organization (WHO) [1].

All currently available antipsychotic drugs exert their primary therapeutic effects by acting directly or indirectly on dopaminergic signaling. Despite differences in receptor-binding profiles, canonical antipsychotics share a common pharmacodynamic feature, namely dopamine D2 receptor (D2R) occupancy, either as antagonists or partial agonists at D2/D3 receptors [2]. Beyond receptor interaction, a deeper understanding of the effects of these drugs on the micro- and macro-structural neural architecture, through the identification of trans-synaptic targets, currently represents a major challenge [3,4,5]. Although dopaminergic dysregulation remains a cornerstone of schizophrenia pathophysiology, converging evidence has highlighted a central role for glutamatergic signaling abnormalities, particularly within the framework of the N-methyl-D-aspartate receptor (NMDAR) hypofunction hypothesis [6,7,8]. Over the past decades, metabotropic glutamate receptors (mGluRs) have attracted increasing interest due to their involvement in neural circuits positioned at the crossroads of glutamate–dopamine–serotonin triple interplay, which are considered dysregulated in schizophrenia [9].

Within this framework, mGluRs and their associated intracellular signaling partners, including postsynaptic density (PSD) proteins at glutamatergic synapses, have emerged as critical molecular hubs. The PSD is a disk-shaped, electron-dense structure localized at the postsynaptic membrane of glutamatergic synapses, characterized by a tripartite organization, composed of an intricate molecular mesh including receptor adaptors, scaffolding proteins, and structural elements forming a functional switchboard that regulates receptor stability, lateral diffusion within the membrane, receptor internalization, and the propagation of synaptic signals from the cell surface to intracellular compartments [10,11]. These complexes are increasingly viewed as hot spots both for understanding the extended mechanisms of action of currently available antipsychotics, as well as promising molecular targets for the development of novel therapeutic agents [12,13]. Novel strategies aimed at directly modulating PSD proteins have emerged in the last decade. Among these, benzothiazole aniline derivatives such as SPG302 are under clinical evaluation for neurological and psychiatric disorders [14]. Preclinical studies suggest that these compounds may restore dendritic spine density and morphology in hippocampal CA1 neurons by increasing PSD-95 expression at the PSD [15].

In the present review, we focus specifically on the PSD at glutamatergic synapses and its relevance to schizophrenia pathophysiology and treatment. Several core PSD proteins interact directly or indirectly with group I mGluRs, including members of the Homer family, which play a pivotal role in organizing receptor-associated signaling complexes, giving rise to a highly organized “molecular Lego” architecture, in which scaffolding and signaling proteins converge to integrate multiple neurotransmitter inputs, particularly those related to dopamine–glutamate interactions [16].

Refs. [14,15], this topic may hold significant implications for tackling forms of the disease that do not respond to standard treatments, namely treatment-resistant schizophrenia (TRS), whose pathophysiology is likely to differ from that of patients who respond to antipsychotics [17]. TRS affects approximately 30% of subjects with schizophrenia [1,18] and is defined by insufficient clinical response after at least two different antipsychotic trials [17]. Clinically, TRS is associated with severe functional impairment, a higher prevalence of soft neurological signs [19], greater disorganization symptoms [20], altered reward response [21], and cortical–subcortical connectivity [22].

Here, we examine the role of macromolecular complexes formed by mGluRs and PSD proteins in schizophrenia pathophysiology and treatment, addressing the following key questions:

1. What evidence from genomic, post-mortem, and preclinical studies supports the involvement of mGluR1- and mGluR5-associated PSD proteins in schizophrenia?
2. How do the mGluR-associated PSD proteins interact with other PSD components to maintain dendritic structure and synaptic function relevant to schizophrenia?
3. Can alterations in mGluR–PSD protein complexes account for synaptic dysfunction at the microdomain level and its relationship to large-scale connectivity changes and symptom domains?
4. From a therapeutic perspective, how do current antipsychotic treatments affect mGluR-PSD protein complexes, and to what extent might novel non-canonical compounds targeting these complexes provide a complementary strategy to existing antipsychotics?

## 2. Overall Organization and Function of mGluRs Relevant to Schizophrenia Pathophysiology

### 2.1. Overview of mGluRs

mGluRs serve as neuromodulatory L-glutamate/L-aspartate receptors [23,24], regulating synaptic transmission and plasticity via second messenger signaling pathways, including cyclic adenosine monophosphate (cAMP), inositol trisphosphate (IP3), and diacylglycerol (DAG) [25], which lead to the opening of ion channels or other intracellular cascades [26]. Unlike ionotropic receptors, which form an ion channel pore and operate rapidly, mGluRs are indirectly linked to ion channels through signal transduction mechanisms, resulting in a slower activation but more prolonged effects [25,27]. mGluRs are G protein-coupled (GPCRs) and membrane-bound receptors activated by a range of extracellular ligands, including neurotransmitters, small molecules, and peptides [28,29,30,31,32]. Often referred to as rhodopsin-like GPCRs, mGluRs share structural similarities, including an intracellular C-terminal tail, seven hydrophobic transmembrane domains, and an extracellular N-terminal domain [33]. This extracellular region consists of a cysteine-rich domain, which closes around the ligand, inducing conformational changes [34], and a Venus flytrap domain (VFD), characterized by two lobes, separated by a cleft containing the glutamate binding site [35,36].

Based on sequence homology, pharmacology, and signaling pathways, mGluRs are divided into three groups (see Table 1) [37,38]:

(i) Group I includes mGluR1 and mGluR5, which couple to Gq/11 proteins to activate phospholipase C, producing IP3 and DAG mediating postsynaptic signaling [25,39]. These receptors are predominantly expressed at postsynaptic sites, where their activation leads to increased neuronal excitability [40,41,42,43,44]. Furthermore, these receptors are key regulators of synaptic plasticity, influencing both long-term potentiation (LTP) and long-term depression (LTD) at glutamatergic synapses, thereby contributing to enduring modifications in neuronal activity [45].
(ii) Group II includes mGluR2 and mGluR3, which couple to Gi/o proteins to inhibit adenylyl cyclase and reduce cAMP, often acting presynaptically to suppress glutamate release [44,46,47,48]. This inhibitory mechanism operates across various synapses, including excitatory (glutamatergic), inhibitory (γ-aminobutyric acid, GABA-ergic), and neuromodulatory (monoamines, acetylcholine, or neuropeptides) synapses [45,49].
(iii) Group III consists of mGluR4, mGluR6, mGluR7, and mGluR8, also Gi/o-coupled, primarily presynaptic autoreceptors that modulate neurotransmitter release via cAMP inhibition, except mGluR6, which is expressed at postsynaptic sites but restricted to the retina [44,50,51,52,53,54].

**Table 1:** The table provides information about key functions and knockout phenotypes of mGluR subtypes. Abbreviations: mGluR, metabotropic glutamate receptor; LTP, Long-Term Potentiation; LTD, Long-Term Depression; CA3, Cornu Ammonis 3; GABA, Gamma-Aminobutyric Acid.

mGluR Group	mGluR Subtype	Key Functions	Knockout Phenotypes	Clinical Correlates of Specific mGluR Subtype Dysfunctions
Group I [40,41,42,43,44]	**mGluR1**	Learning and memory, synaptic plasticity (LTP/LTD), cerebellar function, taste	LTP deficits, impaired context-specific learning Sensorimotor gating deficits, characteristic of psychotic disorders Cerebellar gait abnormalities Defective innervation of cerebellar neurons	Ataxia, cognitive impairment
	**mGluR5**	Learning and memory, addiction, motor regulation, metabolism regulation	Sensorimotor gating deficits, characteristic of psychotic disorders Reduced response to cocaine Resistance to high-fat diet-induced obesity	Schizophrenia, addiction, Fragile X syndrome, metabolic disorders
Group II [47,48]	**mGluR2**	LTD at mossy fiber-CA3 synapses, cognitive function	Normal synaptic transmission but impaired LTD Increased cocaine responsiveness Cognitive impairment	Addiction, cognitive dysfunction, psychosis
	**mGluR3**	Astrocyte-mediated neuroprotection, synaptic modulation	Loss of neuroprotection against excitotoxicity	Neuroprotection, excitotoxicity-related disorders
Group III [50,51,55]	**mGluR4**	Motor learning, seizure modulation, spatial memory	Impaired motor learning and spatial memory Modulates GABA(A)-mediated seizure activity Lacks ethanol-induced motor stimulation	Epilepsy, motor coordination disorders
	**mGluR6**	Retinal ON bipolar cell signaling	Delayed ON response to light	Vision disorders
	**mGluR7**	Glutamate regulation (brake mechanism), amygdala-dependent learning	Epileptic phenotype Learning deficits Increased anxiety and depression-related behaviors	Epilepsy, anxiety, depression
	**mGluR8**	Anxiety regulation, metabolism, gut motility	Increased anxiety and weight gain Possible role in gut motility and insulin secretion	Anxiety disorders, metabolic disorders

In summary, Group I mGluRs generally enhance NMDAR function and excitatory signaling, whereas Group II and III mGluRs tend to constrain glutamatergic transmission and excitotoxicity. This functional balance is highly relevant to schizophrenia pathophysiology and provides a rationale for targeting distinct mGluR subtypes in the modulation of synaptic plasticity and network stability.

### 2.2. Trafficking and Clustering of Group I Metabotropic Glutamate Receptors Are Coordinated by PSD Scaffolding Proteins: Mechanisms and Synaptic Implications

Receptor trafficking is essential for ensuring the precise spatiotemporal distribution of membrane receptors, including mGluRs, at the cell surface [56]. Among the mechanisms regulating receptor availability, internalization plays a key role in desensitization by preventing excessive receptor activation. Group I mGluR internalization is rapid and primarily mediated by the recruitment of dynamin and β-arrestins [57,58], which inhibit further G protein signaling and simultaneously act as scaffolding proteins to assemble endocytic adaptor complexes [59,60,61]. Once internalized, mGluR1 and mGluR5 are sorted into endosomal compartments and recycled back to the surface, facilitating resensitization [62,63,64,65,66]. The disruption of these processes may result in receptor mislocalization and aberrant signaling. 

Group I mGluRs interact with downstream signaling molecules through Homer proteins [67,68,69,70,71,72], which function as crosslinkers at the PSD. Homer proteins exist as constitutive (Homer1b/c, Homer2a/b, Homer3) and inducible (Homer1a and Ania-3) isoforms [10,73,74,75,76]. Long forms contain an N-terminal enabled/vasodilator-stimulated phosphoprotein homology (EVH1) domain that binds the proline-rich motif of Group I mGluRs, and a C-terminal coiled-coil domain allowing multimerization. In contrast, short forms lacking the C-terminal domain act as dominant negative regulators [77,78], disrupting interactions of long forms. Homer1c assists in the clustering of Group I mGluRs at postsynaptic sites, anchoring mGluRs and approximating them to other intracellular effectors such as IP3 receptor and ryanodine receptor 1 (RyR), whereas Homer1b retains mGluR5 within the endoplasmic reticulum [79,80,81,82]. Increased Homer1a expression reduces membrane levels of mGluR1, whereas effects on mGluR5 are divergent [83,84]. Accordingly, the Homer1a/Homer1b ratio regulates dendritic spine architecture and could influence the direction of mGluR-mediated plasticity [85,86,87,88]. Specifically, an increase in Homer1b enhances mGluR5 signaling, whereas elevated Homer1a levels shift signaling toward mGluR1-dependent pathways [83].

The spatial arrangement of mGluRs relative to NMDARs and α-amino-3-hydroxy-5-methyl-4-isoxazole propionate receptors (AMPARs) is crucial for synaptic strength. The interaction between mGluR5 and NMDARs is regulated by Homer or PSD-95, which act as bridges. Disruption of this interaction leads to aberrant mGluR5 mobility and increased co-clustering with NMDARs [89], and is associated with cognitive impairment, reduced synaptic plasticity, and impaired NMDAR function. Restoration of correct mGluR5–Homer coupling rescues these abnormalities, highlighting the critical role of this interaction in maintaining synaptic strength [89].

Super-resolution imaging techniques (gated stimulated emission depletion—gSTED, single-molecule localization microscopy—SMLM) reveal that mGluR5 forms nanoscale clusters within 200 nm of the PSD border, avoiding direct entry into the PSD core [90]. These nanodomains occupy a median area of 6.0 × 10^3^ nm^2^ and exhibit confined diffusion dynamics. Homer1b favors the confinement of mGluR5 within these clusters, limiting their lateral movement across the membrane [91]. Unlike AMPARs (e.g., GluA2), which colocalize with PSD markers, mGluR5 shows minimal overlap with core PSD proteins, an exclusion mediated by its C-terminal domain, which prevents synaptic entry even under conditions of forced scaffold interaction [90]. Super-resolution imaging confirms that mGluR1 also clusters within 100–300 nm of PSD borders, often forming stable nanodomains (~5–10 × 10^3^ nm^2^) with confined diffusion, as detected in cerebellar parallel fiber-Purkinje cell and hippocampal synapses [92]. Homer1b/c similarly restricts mGluR1 lateral mobility by cross-linking to Shank-profilin scaffolds, thereby maintaining perisynaptic positioning essential for detecting glutamate spillover. During high-frequency firing, low extrasynaptic levels (~100 nM–1 μM) from adjacent active synapses can evade rapid transporter clearance, triggering IP3/Ca^2+^ signaling that supports LTD and motor learning [92].

Shank family further contributes to the clustering of Group I mGluRs, coupling them with other PSD components due to a PDZ domain that can bind the C terminus of Group I mGluRs. Shank proteins serve as critical adapters that physically bridge Homer-associated mGluRs with PSD-95/NMDAR complexes [93]. Studies conducted in COS cells have demonstrated that Shank clusters with Homer1b and, together with guanylate kinase-associated protein (GKAP), mediates the co-clustering of Homer and PSD-95 [93]. This macromolecular complex, comprising Homer/Shank/GKAP/PSD-95, links Group I mGluRs to NMDAR scaffolds, potentially coordinating signaling crosstalk during synapse maturation [94], by facilitating mGluR recruitment into developing synapses anchored to pre-existing NMDAR cores, mirroring the ontogenetic sequence where NMDAR transmission precedes AMPAR and mGluR integration [93]. In fact, while mGluRs are present from birth, their synaptic activation lags behind NMDAR currents, over the progressive maturation of glutamatergic signaling at corticothalamic synapses, where NMDAR predominance yields gradually to AMPAR and mGluR contributions [95].

Homer–Shank complex not only supports the perisynaptic localization of Group I mGluRs in proximity to NMDARs [41], but this spatial arrangement could enhance the cooperative activation of phospholipase C (PLC) and protein kinase C (PKC) pathways and converge on Ca^2+^ signaling through Ca^2+^-induced Ca^2+^-release (CICR) mechanisms involving IP3 receptors (IP3R) and RyRs [96,97,98,99]. These processes are mediated by the multimerization of constitutively expressed Homer isoforms, which link the Shank/GKAP/PSD-95 complex to IP3R/RYR and mGluRs [71,93]. Such macromolecular assemblies combining mGluRs, NMDARs, and CICR channels may underlie synergistic effects of Group I mGluRs on NMDAR-dependent LTP, providing a mechanistic framework for the attenuated synaptic responses observed in Group I mGluR mutant mouse models [100,101,102,103,104,105].

Notably, pharmacological treatments, including antipsychotics, as well as stress exposure, modulate Homer1a and PSD protein expression in a dose-, receptor-, and time-dependent manner [74,87,106,107,108,109].

In this context, SPG302, a third-generation benzothiazole derivative currently under evaluation in a randomized, placebo-controlled, double-blind phase 2 clinical trial in patients affected by schizophrenia [14], with an estimated completion in February 2026, has been shown to restore phrenic motor neuron activation in a rat model of cervical spinal cord injury by enhancing spared local synaptic connections [110]. This effect is likely mediated by an upregulation of PSD proteins, including PSD-95. By enhancing PSD-95 expression, SPG302 may potentiate receptor–scaffold protein interactions, and promote the formation of Group I mGluR signaling microdomains, thereby facilitating synaptic remodeling and more efficient glutamatergic transmission.

## 3. Role of mGluRs in Synaptic Plasticity

### 3.1. Mechanisms of Synaptic Plasticity: Long-Term Potentiation (LTP) and Long-Term Depression (LTD)

Synaptic plasticity refers to activity-dependent, long-lasting changes in synaptic strength, classically expressed as LTP or LTD, induced by high- or low-frequency stimulation patterns, respectively. These mechanisms are essential for the storage of information in neural systems, contributing to memory encoding and consolidation as well as the optimization of neural activity and remodeling of circuits [111,112,113].

It is well known that AMPAR trafficking in postsynaptic neurons is required for the expression of LTP and LTD [114,115,116]. When glutamate binds to receptors, the opening of the pore allows the influx of Na^+^ ions (along with K^+^ outflow) and depolarizes the postsynaptic membrane. AMPARs also allow Ca^2+^ influx by engaging Ca^2+^-dependent signaling events. Thus, the specific localization and regulation of the number of AMPARs at the cell surface are crucial for synaptic plasticity [117,118].

NMDARs and mGluRs are both essential for bidirectional synaptic plasticity at different stages, exhibiting opposite yet complementary roles in plasticity [114,119]. While Group I mGluRs, such as mGluR5, promote the synthesis of proteins involved in LTP and LTD, NMDAR stimulation suppresses the translation of these proteins. These opposing effects on bioenergetics indicate a coordinated function in preserving neural activity [120].

Group I mGluRs, enriched at peri- and postsynaptic sites, regulate AMPAR trafficking via G protein-coupled pathways. Importantly, these effects are mediated by interactions with PSD proteins that function as molecular adaptors linking mGluRs to downstream trafficking machinery. Tamalin, a PSD protein with a C-terminal PDZ binding motif, interacts with Group I mGluRs [121], and its knockdown impairs mGluR-dependent AMPAR internalization, disrupting synaptic strength and plasticity [122]. Similarly, Norbin binds the membrane-proximal region of mGluR5, regulating agonist-induced endocytosis and facilitating protein kinase A (PKA)-mediated phosphorylation of AMPARs [123]; its loss inhibits Group I mGluR-dependent AMPAR endocytosis, affecting synaptic plasticity [123]. 

Moreover, mGluRs modulate neuronal activity via a mechanism known as mGluR-dependent LTD (involving mGluR1/5), leading to Ca^2+^-dependent synaptic weakening [63,124]. Evidence suggests that mGluR5 plays a multifaceted role in inducing and maintaining LTD, acting both independently and synergistically with NMDARs. When mGluR5 is activated by the glutamate binding, it initiates an intricate GPCR signaling cascade, ultimately leading to the dephosphorylation and internalization of AMPARs, weakening the synaptic efficacy. Although both NMDARs and mGluRs promote AMPAR internalization during LTD, they target different AMPAR populations: NMDAR-dependent LTD preferentially affects rapidly cycling AMPARs, whereas mGluR-dependent LTD targets more stable AMPAR populations. This segregation of signaling pathways enables precise control of synaptic strength. 

**Figure 1:**
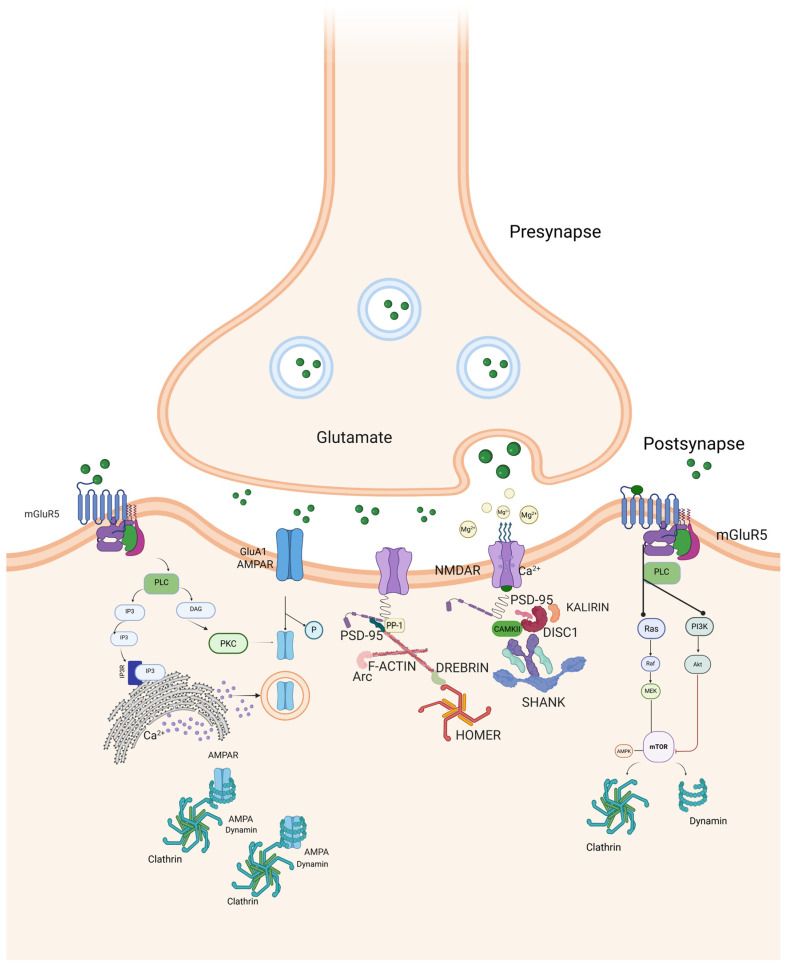
The arrangement of the different glutamatergic receptors at the postsynaptic level and the various pathways activated by mGluRs and NMDAR during LTD. Abbreviations: Akt = Protein kinase B (PKB); AMPA = α-amino-3-hydroxy-5-methyl-4-isoxazolepropionic acid; AMPAR = α-amino-3-hydroxy-5-methyl-4-isoxazolepropionic acid Receptor; CaMKII = Calcium/calmodulin-dependent protein Kinase II; Clathrin = Clathrin coat protein; DAG = DiacylGlycerol; DISC1 = Disrupted In Schizophrenia 1; F-ACTIN = Filamentous Actin; GluA1= Glutamate receptor A1 subunit; IP3 = Inositol trisphosphate; IP3R = Inositol trisphosphate receptor; AMPK= AMP-activated protein kinase; KALIRIN = Kalirin Rho guanine nucleotide exchange factor; MEK = Mitogen-activated protein kinase kinase; mGluR5 = metabotropic Glutamate Receptor 5; mTOR = mammalian Target Of Rapamycin; NMDAR = N-methyl-D-aspartate Receptor; PI3K = Phosphatidyl Inositol 3-Kinase; PKC = Protein Kinase C; PLC = PhosphoLipase C; PP-1 = Protein Phosphatase 1; PSD-95 = PostSynaptic Density protein 95; Raf = Rapidly Accelerated Fibrosarcoma kinase.

mGluR5 signaling can activate the mammalian target of rapamycin complex (mTORC1), which increases the synthesis of proteins involved in receptor trafficking, such as dynamin and clathrin [124,125] (Figure 1), contributing to the maintenance of LTD [126,127]. mGluR5 and mTOR are also involved in synaptic downscaling, a process in which synaptic strength is reduced to maintain the network’s overall stability, especially in conditions of network overactivity or hyperexcitability [124,125,128,129]. Through these mechanisms, mGluR-dependent signaling at the PSD plays a fundamental role in shaping activity-dependent synaptic plasticity. Thus, beyond their role in plasticity, mGluRs also support neuronal resilience. mGluR5 activation can reduce NMDAR-induced neurotoxicity and preserve mitochondrial function by disrupting the NMDAR–PSD-95 complex.

Similarly, mGluR7 modulates NMDAR-mediated currents via the cofilin/actin signaling pathway, contributing to neuroprotection under excitotoxic conditions [130]. These mechanisms highlight how mGluR-dependent signaling at the PSD not only shapes activity-dependent plasticity but also safeguards neurons against excitotoxic damage.

### 3.2. mGluR 1/5 and Metaplasticity

Metaplasticity is the homeostatic mechanism by which prior neuronal activity modulates the threshold, direction, or magnitude of subsequent synaptic plasticity, representing a higher-order regulatory mechanism that ensures synaptic stability and adaptability over time [131]. Rather than encoding information directly, metaplasticity defines the “rules” for future plastic changes, preventing saturation of synaptic strength and maintaining network homeostasis. A priming stimulus can modify neurotransmitter release or signaling, altering the functional state of neurons and synapses for minutes to hours, thereby setting the physiological state for subsequent plasticity [132].

In this context, a well-characterized example of group I mGluR involvement in metaplasticity is the observation that mGluR5 activation has been shown to enhance the sensitivity of NMDARs to glutamate [133], through the phosphorylation of the GluN2B subunit, thus making them more responsive to low-frequency stimulation. An in vivo study demonstrated that mGluR5, by enhancing NMDAR function, lowers the threshold for LTD induction in the hippocampus and increases resistance to NMDAR antagonists, such as D-2-amino-5-phosphonopentanoic acid [133].This means that weaker synaptic stimuli can induce LTD more effectively (see Table 2). In this way, mGluRs’ activation, by integrating prior patterns of synaptic activity, translates them into long-lasting modifications of synaptic signaling competence, and alters the synapse’s future responsiveness to identical stimuli.

Also, activity-dependent remodeling of the PSD, such as shifts in the balance between short and long Homer isoforms, can alter mGluR coupling to intracellular effectors, effectively reprogramming downstream signaling pathways and biasing future plasticity outcomes. Through such mechanisms, mGluR–PSD interactions encode a molecular “memory” of prior synaptic activity, stabilizing changes in receptor localization, signaling efficiency, and Ca^2+^ microdomain organization. In this context, several pieces of evidence show that mGluRs are able to modulate plasticity over longer timescales [134], helping to gate or fine-tune future plasticity responses based on previous activity levels by modulating AMPAR availability, engaging protein synthesis, or kinase activation [135,136,137].

Presynaptic mGluRs also contribute to metaplastic regulation, as the internalization of mGluR7, a Group III mGluR subtype, has been shown to unmask a metaplastic state in the hippocampus. The internalization modifies the synaptic response to subsequent stimuli [138]. All these mechanisms create a sort of “synaptic tag” that prepares the neuron for lasting plasticity changes based on past patterns of activity.

Additional metaplastic mechanisms involve retrograde signaling mediated by endocannabinoids and nitric oxide (NO), which are synthesized postsynaptically following mGluR activation and act on presynaptic terminals to suppress neurotransmitter release, including that of glutamate [114,139]. By modulating presynaptic release probability in an activity-dependent manner, these pathways further shape the synapse’s future plastic potential.

Collectively, these processes support the view that metaplasticity arises from the coordinated action of mGluRs and PSD-associated molecular networks, which together regulate synaptic learning rules and ensure adaptive plasticity across varying temporal and activity scales.

**Table 2:** This table summarizes the different mechanisms of mGluR-mediated LTD compared with those of NMDAR-mediated LTD. The diverse activation mechanisms reflect the distinct roles of these two forms of LTD in homeostatic synaptic plasticity. In mGluR-LTD, mGluR5 signaling activates mTORC1, which promotes the synthesis of proteins required for synaptic remodeling. These proteins include clathrin, dynamin, and other scaffolding proteins involved in endocytosis and degradation of AMPARs. Abbreviations: mGluR, metabotropic Glutamate Receptor; LTD, Long-Term Depression; NMDAR, N-Methyl-D-Aspartate Receptor; GPCR, G Protein-Coupled Receptor; PLC, Phospholipase C; IP3, Inositol trisphosphate; CaMKII, Ca^2+^/Calmodulin-dependent Protein Kinase II; PKC, Protein Kinase C; PP1, Protein Phosphatase 1; PP2B, Protein Phosphatase 2B (Calcineurin); mTOR, Mammalian Target of Rapamycin; AMPAR, α-amino-3-hydroxy-5-methyl-4-isoxazolepropionic acid receptor.

	mGluR-Dependent LTD	NMDAR-Dependent LTD
Receptor Activation	Activation by glutamate [63]	Activation by glutamate combined with postsynaptic depolarization [114]
Primary signaling mechanism	GPCR activation leading to PLC signaling and IP3-mediated Ca^2+^ release from intracellular stores (e.g., endoplasmic reticulum) [92]	Direct Ca^2+^ influx through NMDAR channels [119]
Intracellular signaling cascades	Activation of PKC via DAG; involvement of CaMKII and protein phosphatases (PP1, PP2B) [92]	Activation of CaMKII and protein phosphatases [140]
AMPAR modulation	AMPAR dephosphorylation and endocytosis; mTOR-dependent protein synthesis contributing to LTD maintenance [122,123,124]	AMPAR dephosphorylation and removal from the postsynaptic membrane [114]
Functional role in synaptic plasticity	Homeostatic synaptic plasticity (scaling down synaptic strength) [125,128]	Activity-dependent synaptic plasticity (fine-tuning synaptic strength) [119]

## 4. The Role of mGluRs in Schizophrenia Pathophysiology

Given mGluRs’ ability to modulate synaptic transmission and plasticity, they may offer a key for understanding the neurobiology of schizophrenia. This section summarizes the available evidence from post-mortem studies, in vivo research, and neuroimaging findings pointing to mGluRs structural or functional alterations in schizophrenia, shedding light on their contribution to the physiopathology of the disorder.

In a post-mortem study, increased levels of mGluR1α mRNA have been observed in the prefrontal cortex (PFC) of schizophrenia patients compared to healthy controls [141]. These differences do not appear to be attributable to antipsychotic medications or other potential confounds, suggesting an upregulation of this receptor subtype in schizophrenia [141]. Accordingly, a Western blot analysis conducted in subjects with schizophrenia also found an increase in mGluR1 and mGluR2/3 immunoreactivity in the PFC, but not in striatal regions [142], while no changes in the expression of mGluR4a or mGluR5 were detected. Studies on mGluR5 expression have revealed more nuanced changes. Of interest, the downregulation of miR-501-3p in schizophrenia patients—observed in monozygotic twins discordant for the disorder [143]—directly elevates mGluR5 expression. This miRNA normally suppresses mGluR5 translation, and its loss leads to unchecked receptor activity [143].

Animal models have been instrumental in elucidating the role of mGluR subtypes in schizophrenia-like behaviors. Elevated mGluR5 in miR-501-3p knockout mice has been found to drive dendritic spine abnormalities, enhanced glutamatergic transmission, and disrupted sociability, memory, and sensorimotor gating, mirroring the core deficits of schizophrenia phenotypes. Thus, it has been argued that excessive mGluR5 signaling destabilizes NMDAR activity, shifting the excitatory-inhibitory imbalance toward excitotoxicity [143].

Group II mGluRs also exhibit alterations, but findings across studies remain inconsistent. Some studies report a decreased binding density of mGluR2/3 in the PFC of schizophrenia patients [144], suggesting a potential reduction in their regulatory role in glutamate transmission, as these receptors negatively regulate glutamate release and counteract excitotoxicity. Others, however, find no significant differences in receptor density [145,146,147]. It has been reported that a negative correlation exists between mGluR2/3 density and age at death, which might indicate a progressive vulnerability to glutamatergic dysregulation over time [145].

mGluR2 interacts with the serotonin 5-hydroxytryptamine 2A receptor (5-HT_2A_R), through specific transmembrane helix domains, forming functional complexes in the brain cortex [144]. This 5-HT_2A_R–mGluR2 complex elicits unique cellular responses upon stimulation by hallucinogenic drugs [144]. Notably, activation of mGluR2 suppresses hallucinogen-specific signaling and behavioral responses. In post-mortem brain tissue from untreated individuals with schizophrenia, an imbalance in receptor expression is observed, with 5-HT_2A_R being upregulated and mGluR2 downregulated—a pattern that may contribute to psychosis vulnerability [144]. Maternal stress (psychological or immune) during mouse pregnancy induces lasting cortical changes in offspring, including downregulation of mGluR2s and upregulation of 5-HT_2A_R in the frontal cortex [148]. Behaviorally, these changes manifest as increased sensitivity to hallucinogenic drugs targeting 5-HT_2A_R and reduced efficacy of antipsychotic-like compounds acting on mGluR2/3 [148]. The inverse relationship between mGluR2 and 5-HT_2A_R creates a cortical imbalance: reduced mGluR2 activity fails to counteract 5-HT_2A_R-driven excitatory signaling, underlying aberrant perception, cognition, and psychosis-like behaviors.

Regarding genetic studies, single-nucleotide polymorphisms (SNPs) in genes encoding mGluRs have been associated with schizophrenia. For instance, a mutation affecting mGluR5 signaling pathways have been implicated in cognitive deficits observed in patients [149,150]. A genome-wide association study (GWAS) identified the GRM7 locus as a potential schizophrenia risk region and further demonstrated that specific GRM7 variants, such as rs1516569 (OR = 0.95, *p* < 3.47 × 10^−4^), are associated with disease susceptibility, while rs9883258 (OR = 0.84, *p* = 2.18 × 10^−3^) and other polymorphisms (rs779746, rs480409, rs78137319, rs1154370) were linked to differential treatment responses across seven commonly used antipsychotics in a large Chinese Han sample [151].

Moreover, a potential link between SNPs in metabotropic glutamate receptor 3 gene (GRM3), encoding mGluR3, and schizophrenia has been hypothesized. This association was further supported by a large-scale genome-wide association study, although the findings remain conflicting [145,152,153,154,155,156,157,158]. Many GRM3 polymorphisms linked to schizophrenia are in non-coding regions, leaving the precise biological mechanism unclear. Some evidence suggests a role in treatment response, as specific GRM3 variants have been associated with improvements in negative symptoms following olanzapine therapy [159]. Egan et al. proposed a possible pathway through which GRM3 polymorphisms might influence glutamatergic transmission and increase schizophrenia risk [153]. They reported that an intronic variant of GRM3 was linked to poorer cognitive performance on tasks assessing prefrontal and hippocampal function—key domains affected in schizophrenia. Moreover, post-mortem analysis of the human PFC revealed that carriers of this GRM3 variant exhibited lower mRNA levels of excitatory amino acid transporter 2 (EAAT2), a glial glutamate transporter essential for synaptic glutamate clearance. This finding suggests that schizophrenia pathophysiology may involve altered mGluR3 transcription/expression and impaired glutamate neurotransmission, possibly due to reduced EAAT2 expression [153]. GWAS have also revealed that many schizophrenia risk loci encode proteins localized at the PSD, including scaffolding proteins such as PSD-95, Homer1, and Tamalin, as well as NMDAR subunits [160]. Variants in these genes may disrupt mGluRs’ localization and interaction with PSD scaffolds, impairing AMPAR trafficking, NMDAR-mGluR co-clustering, and activity-dependent synaptic plasticity, ultimately contributing to cognitive deficits and disease susceptibility.

Neuroimaging techniques such as magnetic resonance spectroscopy (MRS) have provided non-invasive methods to study glutamatergic alterations in vivo. Elevated levels of glutamate + glutamine (Glx) have been reported in several brain regions of schizophrenia patients, including the basal ganglia, medial temporal lobe, and anterior cingulate cortex [161]. These findings suggest hyperactive glutamatergic transmission, which may underlie excitotoxic damage observed in chronic cases.

Therefore, post-mortem studies reveal altered expression patterns of group I and II mGluRs, while neuroimaging highlights widespread disruptions in glutamate metabolism across key brain regions. In vivo research underscores the behavioral consequences of these changes and identifies potential genetic underpinnings. Together, these findings suggest that targeting mGluRs could offer novel therapeutic avenues for treating schizophrenia symptomatology. While convergent evidence from post-mortem, genetic, animal (knockouts mimicking gating deficits), and imaging studies implicates mGluR-PSD alterations in schizophrenia, findings are largely correlative. Causality remains unproven due to confounds (e.g., antipsychotics, small samples); mechanistic links to symptoms require longitudinal/clinical validation. Future research should focus on refining our understanding of receptor-specific changes and developing personalized interventions based on individual glutamatergic profiles.

## 5. mGluRs as Druggable Targets: Insight into Canonical and Novel Antipsychotic Compounds

### 5.1. Differential Regulation of mGluR Activity by Typical and Atypical Antipsychotics Due to a Trans-Synaptic Effect

Antipsychotics primarily target the striatal dopaminergic system through D2R antagonism and exhibit minimal affinity for glutamatergic receptors. However, growing evidence suggests that antipsychotic treatments appear to indirectly exert their effects, at least in part, by modulating mGluR activity via interactions with PSD proteins, fine-tuning glutamatergic transmission, and the excitatory-inhibitory balance. This effect likely arises from the interplay between D2R and D1R with PSD proteins—including Homer1a, Homer 1b/c, Norbin, Shank, PSD-95—and particularly group I mGluRs [134,162,163]. Dopaminergic activity has been shown to influence the trafficking and localization of mGluRs at synaptic membranes. For instance, D2Rs are responsible for Fyn-mediated tyrosine phosphorylation and synaptic redistribution of mGluR5. In fact, D2R signaling tonically inhibits Fyn kinase activity in striatal neurons, with pharmacological blockade of D2Rs, as obtained by antipsychotics, triggering Fyn-dependent tyrosine phosphorylation of mGluR5 and its subsequent insertion into synaptic membranes [164]. On the other hand, D1Rs regulate mGluR post-translational modifications via serine phosphorylation. D1R is involved in downstream MAP kinase activation, which phosphorylates mGluR5 at serine 1126, affecting its affinity for Homer1b/c, which in turn interacts with Shank and PSD-95, stabilizing receptor insertion into membranes and its localization in presynaptic zones near NMDARs [165]. Therefore, dopamine stabilizes the signaling properties of group I mGluRs, with dopaminergic input loss (via reserpine) disrupting this balance through two divergent effects: (i) a reduction in mGluR1-mediated depolarization in striatal neurons; (ii) enhanced mGluR5 responsiveness, a suggested compensatory mechanism triggered by dopamine depletion [166].

**Figure 2:**
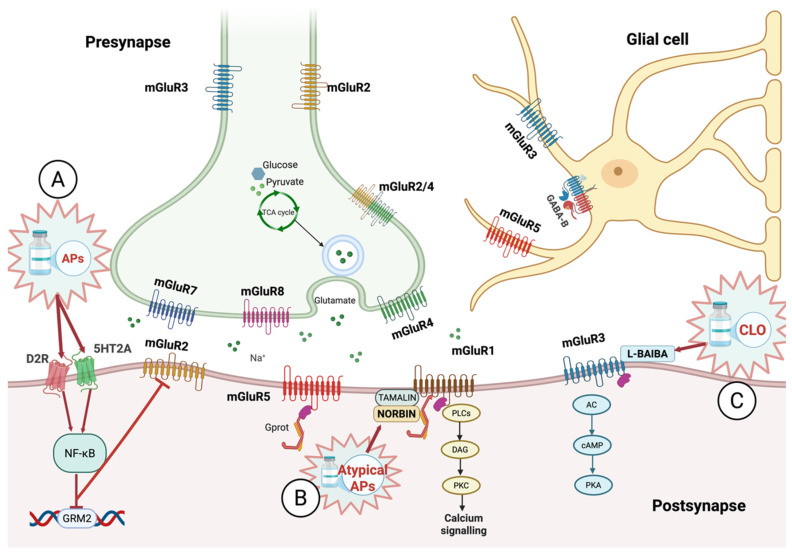
(**A**) Antipsychotics acting on D2 or 5HT2A receptors inhibit the NF-κB signaling pathway, resulting in a downregulation of GRM2. (**B**) Atypical antipsychotics indirectly modulate mGluR1 and mGluR5 and intracellular calcium via PSD proteins, such as Norbin. (**C**) mGluR3s, expressed in both neurons and glial cells, are targeted by clozapine via the metabolite L-BAIBA and regulate cAMP/PKA signaling. Abbreviations: APs: Antipsychotic drugs; 5HT2A: 5-hydroxytryptamine receptor 2A; AC: Adenylate cyclase; cAMP: Cyclic adenosine monophosphate; CLO: Clozapine; D2R: Dopamine D2 receptor; DAG: Diacylglycerol; GABA-B: Gamma-aminobutyric acid type B receptor; Gprot: G protein; GRM2: Glutamate receptor metabotropic 2; HOMER: Homer scaffolding protein family; IP3R: Inositol trisphosphate receptor; L-BAIBA: L-beta-aminoisobutyric acid; mGluR1: Metabotropic glutamate receptor 1; mGluR2: Metabotropic glutamate receptor 2; mGluR2/4: Metabotropic glutamate receptor 2/4; mGluR3: Metabotropic glutamate receptor 3; mGluR4: Metabotropic glutamate receptor 4; mGluR5: Metabotropic glutamate receptor 5; mGluR7: Metabotropic glutamate receptor 7; mGluR8: Metabotropic glutamate receptor 8; NF-κB: Nuclear factor kappa-light-chain-enhancer of activated B cells; NMDAR: N-methyl-D-aspartate receptor; PKA: Protein kinase A; PKC: Protein kinase C; PLCs: Phospholipase C enzymes; PP1: Protein phosphatase 1; PSD-95: Postsynaptic density protein 95.

Chronic dopamine receptor blockade by antipsychotics can modify mGluR1/5 signaling, inducing long-term changes in synaptic plasticity. Antipsychotics differentially regulate mGluR modulators like Homer1a and Norbin; for example, aripiprazole shows a distinct temporal regulation in the nucleus accumbens compared to haloperidol (Figure 2). In a perinatal phencyclidine (PCP) model of schizophrenia, adult PFC shows increased Homer1b/c, Norbin, and juvenile dimeric mGluR5, with decreased mGluR1α dimers [153]. These findings suggest that antipsychotics may exert therapeutic effects by compensating for mGluR alterations, modulating the expression of PSD proteins. This temporal modulation reveals a dynamic “Lego-like” hierarchy of PSD rearrangements under antipsychotic exposure. Acute D2R blockade (e.g., eticlopride within hours) triggers Src family kinase (SFK/Fyn)-mediated tyrosine phosphorylation of mGluR5, promoting its synaptic insertion and lateral mobility in the striatum [164]. Sub-chronic aripiprazole (1 week) upregulates Homer1a in the nucleus accumbens shell, disrupting long-form Homer1bc multimers and shifting mGluR coupling toward perisynaptic reconfiguration. Chronic regimens (e.g., 10 weeks) elevate Norbin and Homer1a/bc ratios, stabilizing spine remodeling via enhanced mGluR5 clustering [167]. Super-resolution imaging (gSTED/SMLM) confirms Homer1b confines mGluR5 to perisynaptic nanodomains (diffusion restricted within 200 nm of PSD border, median cluster area ~60 nm^2^), with isoform shifts dictating speed: rapid minutes for short Homer1a disruption vs. slower hours for long-form multimerization [90]. In summary, inducible immediate-early proteins such as Homer1a can be expressed within minutes to hours, competitively displacing long Homer isoforms (expressed constitutively and not activity-induced in the short term) from mGluR5 and uncoupling receptors from Shank-based supercomplexes. This early phase is followed by a slower reassembly stage, during which scaffold hierarchies are re-established and receptor mobility is re-constrained. Such temporally ordered rearrangements suggest that drug-induced modulation of mGluR signaling may first destabilize protein assemblies before promoting the formation of more adaptive synaptic configurations.

Notably, the ability of antipsychotics to influence glutamatergic transmission extends beyond group I mGluRs, as emerging evidence indicates that mGluR2/3 and group III mGluRs may also play a role. In this regard, a preclinical study demonstrated that the administration of the mGluR2/3 antagonist LY-341495 induced deficits in habituation to acoustic startle or odor-elicited orienting response in male Naval Medical Research Institute (NMRI) mice, mimicking cognitive abnormalities observed in schizophrenia. Of interest, these deficits were reversed by antipsychotic treatment, including haloperidol (0.1–0.3 mg/kg), clozapine (3–10 mg/kg), risperidone (0.03–0.1 mg/kg), olanzapine (1–3 mg/kg), aripiprazole (1–3-10 mg/kg), and amisulpiride (10 mg/kg) [168]. These results suggest that antipsychotics may exert additional modulatory actions on mGluR2/3, potentially contributing to their ability to restore cognitive functions in schizophrenia.

Clozapine has also been shown to modulate mGluR2 and mGluR3 levels in schizophrenia patients, as revealed by post-mortem studies, a finding replicated in preclinical models for mGluR2, but not for mGluR3 [169]. Both mGluR2 and mGluR3 have been found reduced in dorsolateral PFC of schizophrenia patients, as demonstrated by a post-mortem study, suggesting an insufficient cortical availability of group II mGluRs associated with schizophrenia. While chronic antipsychotic treatment normalizes mGluR3 levels, arguably through an epigenetic control mechanism of the expression of the receptor, generating a greater load of permissive histone posttranslational modifications at the mGlu3R gene, it does not appear to affect mGluR2 levels. This effect has been observed following treatment with various antipsychotics, including amisulpiride, aripiprazole, clotiapine, clozapine, haloperidol, levomepromazine, olanzapine, paliperidone, quetiapine, risperidone, sulpiride, and ziprasidone [169].

Chronic administration of clozapine, as well as of other atypical antipsychotics binding to 5-HT_2A_R, was found to reduce the expression of Inhibitor of kappa B alpha (IκBα), a Nuclear Factor kappa-light-chain-enhancer of activated B cells (NF-κB) suppressor, via Extracellular Signal-Regulated Kinases 1/2 (ERK1/2) hypophosphorylation. This process increases NF-κB levels, which, in turn, elevates histone deacetylase 2 (Hdac2) levels, ultimately downregulating mGluR2 expression (Figure 2) [170,171]. Thus, we can assume that chronic atypical antipsychotic exposure facilitates mGluR3 expression while simultaneously suppressing mGluR2, possibly due to differences in promoter sensitivity to histone modifications and transcription factor activity.

A preclinical pharmacological study investigating the effects of clozapine on thalamocortical glutamatergic transmission provides experimental support for a potential modulatory role of presynaptic group III mGluRs in schizophrenia. Interestingly, the study showed that clozapine reduced NMDA antagonist–induced hyperglutamatergic activity in the medial PFC via activation of presynaptic group III receptors, highlighting their possible contribution to the drug’s efficacy in treatment-resistant cases [172]. These receptors could be recruited pharmacologically as a modulatory brake in a hyperglutamatergic state.

Furthermore, administration of clozapine was shown to dose- and time-dependently increase basal L-glutamate and D-serine release from prefrontal astrocytes, an effect mediated in part by group III mGluR activity [173], highlighting a complex interplay between glial release, presynaptic regulation, and mGluR signaling. Another report has demonstrated that clozapine increases the synthesis of L-β-aminoisobutyric acid (L-BAIBA) in the hypothalamus, a molecule capable of activating glycine, GABA-A, and GABA-B receptors [174] (Figure 2). While clozapine does not directly bind these amino acid-modulated receptors, L-BAIBA activates GABA receptors and presynaptic group III mGluRs in astrocytes, contributing to the modulation of tripartite synaptic transmission. Taken together, these findings indicate that clozapine’s effects on NMDAR-dependent glutamatergic signaling may involve presynaptic group III mGluRs. Although not directly part of PSD complexes, their presynaptic and astroglial modulatory actions suggest that they can participate in the regulation of glutamatergic states, supporting a potential role in the pathophysiology of schizophrenia and in its treatment.

These findings reinforce the idea that clozapine’s therapeutic effects extend beyond dopamine antagonism, involving a wide range of neurochemical pathways that may affect mGluRs’ activity, contributing to its unique efficacy in TRS. Notably, the role of mGluR hypofunction in exacerbating schizophrenia symptoms is further supported by evidence of increased levels of mGluR3 and mGluR5 antibodies in a sample of TRS patients who were not receiving clozapine, compared to both non-TRS and TRS subjects treated with clozapine [175]. These findings highlight a potential role for clozapine in preventing hyperglutamatergic states probably due to immune-mediated mechanisms.

These findings highlight the complex interplay between the dopaminergic and glutamatergic systems in clinical conditions. The different effects on signaling and gene expression of mGluRs by typical and atypical antipsychotics suggest potential avenues for developing therapeutic strategies, targeting glutamatergic neurotransmission, with mGluRs as candidate pharmacological targets.

### 5.2. Targeting mGluRs Modulation in Schizophrenia Treatment: Challenges and Opportunities

According to the “NMDAR hypofunction hypothesis” of schizophrenia [176], both positive and negative/cognitive symptoms are linked to increased glutamatergic projection firing. This is proposed to result in excessive glutamate tone, potentially leading to overstimulation of the mesolimbic dopamine pathway and overinhibition of the mesocortical dopamine pathway [177]. Within this framework, Group I receptors, which are largely expressed at postsynaptic sites, may amplify NMDAR-mediated calcium influx, putatively involved in excitotoxicity. On the other hand, group II and group III mGluRs, detectable at both presynaptic and postsynaptic sites of these hyperactive glutamatergic neurons, have been shown to counteract NMDAR activation [177]. At presynaptic sites, they function as autoreceptors regulating glutamate release [177], while at postsynaptic sites, specifically for mGluR2s, they appear to modulate the responses induced by atypical antipsychotics [48,178,179,180].

**Figure 3:**
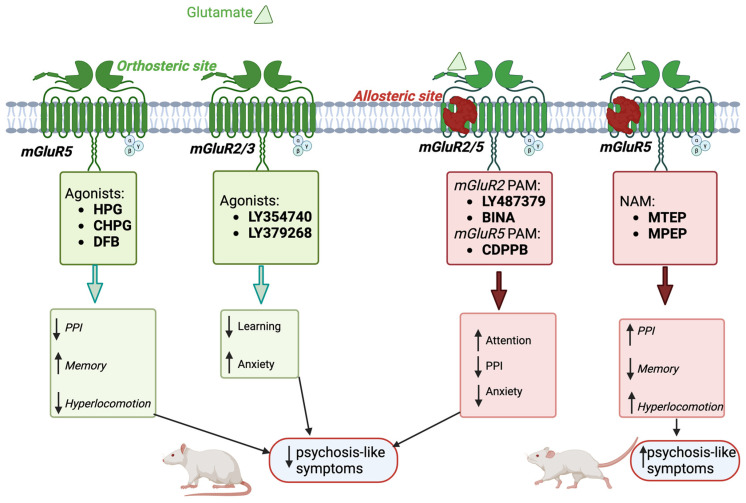
Allosteric (red) and orthosteric (green) modulators of mGluRs and potential implications for schizophrenia, as inferred from animal models. Abbreviations: BINA: N-(4-(2-Methoxyphenoxy)phenyl)-N-(2,2,2-trifluoroethylsulfonyl)piperidine; CDPPB: 3-Cyano-N-(1,3-diphenyl-1H-pyrazol-5-yl)benzamide; CHPG: (RS)-2-Chloro-5-hydroxyphenylglycine; DFB: 3,5-Dihydroxyphenylglycine; HPG: (RS)-3-Hydroxyphenylglycine; LY354740: (1S,2S,5R,6S)-2-Aminobicyclo [3.1.0]hexane-2,6-dicarboxylic acid; LY379268: (1R,4R,5S,6R)-4-Amino-2-oxabicyclo [3.1.0]hexane-4,6-dicarboxylic acid; LY487379: Selective positive allosteric modulator of mGluR2; mGluR: Metabotropic glutamate receptor; MPEP: 2-Methyl-6-(phenylethynyl)pyridine; MTEP: 3-((2-Methyl-1,3-thiazol-4-yl)ethynyl)pyridine; NAM: Negative Allosteric Modulator; PAM: Positive Allosteric Modulator; PPI: Pre-pulse inhibition; SCZ: Schizophrenia.

Preclinical evidence suggests mGluRs could represent druggable targets for schizophrenia and TRS [150], given their role in glutamate signaling and interactions with current antipsychotics. These receptors can be targeted via their ‘orthosteric’ site, which is the same site recognized by the endogenous ligands (Figure 3) [181]. However, clinical translation has proven challenging, and the development of mGluR-targeting antipsychotic drugs faces two major challenges: (i) poor subtype selectivity due to high VFD conservation across mGluR subtypes, and (ii) limited blood–brain barrier (BBB) penetration properties [181]. In fact, orthosteric ligands are predominantly polar compounds, typically characterized by negative clogP values, a key parameter reflecting lipophilicity [182]. While this feature confers high aqueous solubility, it is also associated with low predicted bioavailability [182] and poor BBB penetration, thereby limiting the ability of these compounds to reach their central nervous system (CNS) targets. Indeed, orthosteric agonists (listed in Table 3) typically show low molecular weights (~170–240 g/mol) and very low lipophilicity (with XLogP3-AA values generally ranging from about −4 to −1). Despite these unfavorable physicochemical features, all compounds in this class have shown central effects in vivo. This may be explained by high polarity being associated with an increased unbound drug fraction. As a result, only a small proportion of the compound may need to cross the BBB to achieve pharmacological efficacy [183]. However, a major limitation is their poor oral bioavailability due to limited gastrointestinal absorption. This issue might be overcome through the use of dipeptides as prodrugs [184,185], which are transported via the human peptide transporter 1 (PEPT1) [184] and subsequently cleaved by peptidases or proteases to release the active compound [186]. To address these limitations, new studies are currently underway to explore new strategies and make these receptors viable drug targets for the treatment of schizophrenia [187].

Allosteric modulators that bind to the less-conserved heptahelical transmembrane domain (TMD) offer potential advantages [188]. These modulators can act as: (i) positive allosteric modulators (PAMs), which potentiate the agonist-induced receptor responses; (ii) negative allosteric modulators (NAMs), which noncompetitively antagonize agonists; or (iii) silent allosteric modulators (SAMs), which occupy the allosteric site without affecting the agonist responses but possibly blocking the allosteric effects of both PAMs and NAMs [189,190]. Unlike synthetic orthosteric agonists, these molecules preserve the temporal and spatial patterns of receptor signaling, as their effects depend on the presence of endogenous ligands. Furthermore, their non-continuous receptor activation limits the risk of receptor desensitization and/or downregulation [187,191]. This is probably due to a reduction in receptor saturation, a phenomenon also known as “ceiling effect”, which results from cooperative interactions with the orthosteric ligand [192]. Consequently, allosteric modulators should offer a greater capacity to fine-tune physiological responses while minimizing toxicity risks [193]. Additionally, mGluR allosteric modulators exhibit suitable physicochemical properties, including enhanced capacity to cross the BBB, making them attractive candidates for neuropsychiatric drug development [194]. Specifically, these compounds have both a higher molecular weight (~200–450 g/mol) and markedly higher lipophilicity (XLogP3-AA commonly between 2 and 7, as reported by the National Center for Biotechnology Information, PubChem Compound Summary for CID 16062593, (S)-3,4-Dcpg; retrieved 31 January 2026) (please see Table 3). These properties are consistent with an increased potential to cross the BBB [195].

Despite these advantages, several challenges remain in their clinical application. In particular, one major issue is the formation of mGluR heterodimers [195], as well as the phenomenon of “molecular switches,” where structural changes within the compound scaffold impact its pharmacological properties. This can lead to an unexpected shift from NAM to PAM activity (or vice versa) or even to alterations in their receptor-subtype selectivity [196,197,198]. Furthermore, another aspect that may hinder the replicability of the therapeutic effects is the “biased agonism”, which refers to the ability of GPCRs to adopt only a limited subset of conformations in response to certain ligands [199]. Hence, unintended pathway activation or suppression could lead to unexpected side effects or reduced efficacy. For example, while a biased mGluR2 agonist may effectively reduce glutamate release in one neural circuit, it might fail to engage the necessary downstream mechanisms in another, limiting its therapeutic potential for schizophrenia or other CNS disorders. Biased agonism is recognized as a factor that can influence the efficacy and reproducibility of drugs acting on mGluRs [200]. Different ligands can stabilize distinct receptor conformations and preferentially engage specific intracellular pathways, and this signal distortion can vary depending on the cellular context. For mGluR5, this includes differential activation of intracellular Ca^2+^ mobilization, ERK1/2 phosphorylation, and IP1 signaling [201]. For example, early mGluR5 ago-PAMs such as VU29 and CDPPB show greater intrinsic efficacy for ERK1/2 phosphorylation than Ca^2+^ signaling, whereas glutamate shows the opposite pattern [201]. Importantly, compounds with extreme bias profiles, such as VU0424465, have been associated with adverse effects in preclinical studies, suggesting that biased agonism for a given pathway may contribute to greater side effects [201]. Both ligand and system bias can complicate translation, as the signaling profiles observed in recombinant systems may not be preserved in native tissue or in vivo [200]. This context dependence may partly explain why some mGluR modulators show promising preclinical efficacy but limited or inconsistent clinical benefits.

In the following sections, we will review preclinical and clinical findings on mGluR agonism/antagonism and allosteric modulators’ efficacy in schizophrenia.

**Table 3:** mGluR-targeting molecules and their effects in schizophrenia animal models. Molecular weight and XLogP3-AA values are computed by respectively PubChem 2.2 and XLogP3 3.0. EMQMCM, 3-Ethyl-2-methyl-quinolin-6-yl-(4-methoxy-cyclohexyl)-methanone methanesulfonate; HPG, 3-Hydroxyphenylglycine; PFC, Prefrontal cortex; CHPG, (R,S)-2-chloro-5-hydroxyphenylglycine; PPI, prepulse inhibition; CDPPB, 3-Cyano-N-(1,3-diphenyl-1H-pyrazol-5-yl)benzamide; DFB, 3,3-difluorobenzaldazine; MTEP, 3-((2-Methyl-4-thiazolyl)ethynyl) pyridine; MPEP, 2-methyl-6-phenylethynylpyridine; PCP Phencyclidine; OFC, Orbitofrontal Cortex; DCG-IV, (2S,20R,30R)-2-(20,30-dicarboxycyclopropyl)glycine; EPSP, excitatory postsynaptic potentials; DCPG, (S)-3,4-dicarboxyphenylglycine; BINA, Biphenylindanone A.

Receptors’ Group	Receptor Subtype	Agonism/Antagonism	Pretreatment with Glutamatergic Molecules	Molecular Weight (g/mol)	XLogP3-AA	Drug Dose	Species	Animal Model	Effects	Reference
Group I	mGluR1	Antagonist	Pretreatment with EMQMCM	311.4	4.2	1 and 4 mg/kg	Rat	MK-801 (0.2 mg/kg)	No effect on locomotor activity induced by MK-801 administration	[202]
						4 mg/kg	Rat	MK-801 (0.1 or 0.2 mg/kg)	No effect on PPI disruption induced by MK-801 administration	
						5 mg/kg	Rat	MK-801 (0.1 mg/kg)	Enhance in MK-801 induced ataxia	
		PAM	RO 67-7476	319.4	3.6	4 mg/kg	Rat	Poli I:C (8 mg/kg)	Improvements in PPI, novel object recognition, spontaneous alternation and reference memory tests	[203]
	mGluR5	Agonist	HPG	167.16	−1.4	100 and 500 mM	Rat	PCP (pretreatment: 3–15 mg/kg; after HPG: 7.5 mg/kg)	Reduction in PCP-induced increase in extracellular dopamine in PFC	[204]
			CHPG	201.61	−1.4	50 nmol	Mice	Ketamine (20 mg/kg)	Reduction in locomotor hyperactivity, stereotypy, working memory deficits, PPI deficits	[205]
		PAM	CDPPB	364.4	4.4	3 and 10 mg/kg	Rat	MK-801 (0.1 mg/kg)	Normalization of chaotic PFC neural activity	[206]
						10 mg/kg	Rat	Amphetamine (2 mg/kg)	Amphetamine-induced reversal of locomotor activity and amphetamine-induced deficits in impulse inhibition	[207]
						10 mg/kg	Rat	MK-801 (0.1 mg/kg)	Normalization of OFC neuronal activity	[208]
			DFB	244.24	3.5	40 mg/kg	Mice	Ketamine (20 mg/kg)	Reduction in locomotor hyperactivity, incoordination, working memory deficits	[205]
			VU-29	384.4	4.6	30 mg/kg	Rat	Poli I:C (8 mg/kg)	Improvements in PPI, novel object recognition, spontaneous alternation and reference memory tests	[203]
		Antagonist	MTEP	200.26	2.3	5 mg/kg	Rat	MK-801 (0.2 mg/kg)	Worsening in hyperlocomotion, working memory, stereotypy, and spatial learning	[202]
			MPEP	193.24	3.3	10 mg/kg	Rat	MK-801 (0.1 mg/kg)	Increase in dopamine release and cognitive impairment	[209]
						3 mg/kg	Rat	MK-801 (0.1 mg/kg)	Induction of hyperlocomotion, duration of MK-801-induced stereotypies, impairments of spatial working memory and instrumental learning	[209]
						5 mg/kg	Rat	PCP (2.5 mg/kg)	Enhancement of the locomotor activity increased by PCP	[210]
							Rat	D-amphetamine (1 mg/kg)	Inhibition of amphetamine-induced hyperactivity	
						10 mg/kg	Rat	PCP (1.25 mg/kg	Worsening of PCP-induced memory impairment	[211]
Group II	mGluR2/3	Agonist	LY379268	187.15	−4.3	1 mg/kg	Rat	Ketamine (12 mg/kg)	Reduction in ketamine-induced glutamate release and ketamine-evoked behavioral effects (i.e., stereotypy)	[212]
			LY404039	235.22	−4.8	10 mg/kg	Rat	PCP (5 mg/kg)	Attenuation of the disruptive effects of PCP on working memory, stereotypy, locomotion, and cortical glutamate efflux	[213]
							Marmoset	L-DOPA (15/3.75 mg/kg)	Reduction in global dyskinesia, psychosis-like behaviors and global parkinsonism	[214]
			LY354740	185.18	−3.2	10 mg/kg	Rat	MK-801 (0.1 mg/kg)	Normalization of OFC neuronal activity and reduction in stimulatory effect on glutamate release	[208,212,213]
						3.0 mg/kg	Mice	(-)2,5-dimethoxy-4-bro-moamphetamine [(-)DOB]	Block asynchronous release of glutamate and increased EPSPs	[215]
	mGluR2	PAM	BINA	454.6	7.2	3 μM	Mice	5-HT (10 μM)	Blocking of asynchronous release of glutamate and increased EPSPs and	[216]
						65 mg/kg			Increase in PFC cFos expression	
			LY487379	452.4	4.1	32 mg/kg	Mice	Amphetamine (3.2 mg/kg)	Reduction in amphetamine-induced disruption of PPI of the acoustic startle reflex	[217]
	-	Agonist	DCG-IV	203.15	−4.3	100 nM, 200 nM, 500 nM and 5 mM	Rat	-	Inhibition of the EPSP amplitude	[218]
	-	Indirect agonist	ZJ43	304.30	0.2	200 mg/kg	Mice	MK-801 (1 mg/kg)	Reduction in jumping response	[219]
						150 mg/kg		PCP (6 mg/kg)	Decrease in stereotypic motor activity in the open field assay system	
	-	Indirect agonist	N-Acetylcysteine	163.20	0.4	90 mg/kg	Rat	PCP (1 mg/kg)	Reduction in the increase in extracellular glutamate, social withdrawal and working memory deficits	[220]
Group III	mGluR8	Agonist	DCPG	239.18	−2.7	30 mg/kg	Mice	PCP (1–32 mg/kg) Amphetamine (3–30 mg/kg) Amphetamine (10 nmol)	Ineffective in reducing hyperlocomotion /Reduction in hyperlocomotion	[221,222]
	mGluR7	NAM (ADX71743/ MMPIP)	L-Glu	269.14	3.2	5–15 mg/kg i.p. (30 min pre MK-801)	Mice		Reversed PPI/ social/ novel object recognition deficits MK-801-induced;	
								MK-801	Antipsychotic-like activity in social interaction test (5 and 15 mg/kg);	
			L-Glu; MK-801	333.3	3.4	1–15 mg/kg i.p. (30 min pre MK-801)	Mice	MK-801	Reversed PPI disruption/spatial delayed alternation; Reversed MK-801-induced disturbances in novel object recognition	
							Rat	MK-801 SDA T-maze	Reversed PPI disruption (2.5 mg/kg, *p* < 0.05); Reversed MK-801-induced cognitive/working memory deficit (choice accuracy)	

#### 5.2.1. Preclinical Evidence for mGluR Agonism/Antagonism and Allosteric Modulators: From Animal Models to Clinical Potential

Preclinical and clinical evidence supports the observation that mGluR signaling could be considered as a strategy for normalizing glutamatergic signaling in schizophrenia to reduce psychotic symptoms.

Among group I mGluR-targeting compounds, mGluR5 agonists have been the most extensively investigated as a potential therapeutic approach for schizophrenia [223,224] (Table 3). Considering the interplay of mGluR5 and NMDAR, mGluR5 agonism may reduce physiological, behavioral, and cognitive alterations pharmacological models of schizophrenia obtained by NMDAR antagonism. For example, it has been reported that the administration of mGluR5 PAM 3-Cyano-N-(1,3-diphenyl-1H-pyrazol-5-yl)benzamide (CDPPB) reduces ‘chaotic’ prefrontal cortical activity induced by Dizocilpine (MK-801) administration [206]. Additionally, mGluR5 agonists such as 3-Hydroxyphenylglycine (HPG), (R,S)-2-chloro-5-hydroxyphenylglycine (CHPG) and 3,3-difluorobenzaldazine (DFB) have been demonstrated to attenuate the working memory deficits, locomotor hyperactivity, and prepulse inhibition impairments in both PCP- and ketamine-treated rodents [204,205] (Table 3).

On the other hand, mGluR5 antagonists, including 3-((2-Methyl-4-thiazolyl)ethynyl) pyridine (MTEP) and 2-methyl-6-phenylethynylpyridine (MPEP) were found to exacerbate the cognitive and behavioral effects of NMDAR antagonists [202,209,210,211]. However, no clinical data are currently available for schizophrenia patients, and it remains unclear whether the beneficial effects observed in preclinical models can be translated to humans [225] (Table 3).

For Group II mGluRs, the mGluR2/3 agonist LY354740 blocks PCP-induced increases in cortical glutamate efflux, attenuating the disrupting effects on locomotion and working memory in rats [213,226]. Similarly, treatment with the mGluR2/3 agonist LY379268 has been shown to reduce schizophrenia-like behaviors in rodents pretreated with ketamine, and MK-801 [227,228,229,230] (Table 2), and to restore NMDAR expression levels in a two-hit mouse model of schizophrenia [231]. Other two mGluR2-specific PAMs, namely LY487379 and Biphenylindanone A (BINA), exhibited antipsychotic or anxiolytic effects in rodent models of schizophrenia, supporting the role of mGluR2 in mediating the antipsychotic response (Figure 3) [217,232,233,234] (Table 3).

The available preclinical evidence on antipsychotic-like activity of mGlu7R NAMs is still limited. The only currently available ligands are MMPIP and ADX71743, and both compounds have been found to dose-dependently reverse MK-801-induced hyperactivity and deficits in the novel object recognition test. ADX71743 additionally reversed MK-801-induced disruptions in social interaction and PPI, whereas MMPIP improved spatial delayed alternation performance [222]. These findings suggest that mGluR7 may represent a putative target for antipsychotic drug development, although further studies are warranted given the limited number of available ligands.

Despite these promising results, the lack of clinical data introduces uncertainty regarding their translation to human patients.

#### 5.2.2. Clinical Translation of mGluR-Targeting Strategies in Schizophrenia: Challenges and Opportunities

None of the type I mGluR modulators have been tested clinically in patients with schizophrenia, and only limited and inconclusive evidence exists for their use in other psychiatric disorders [235,236,237]. It is worth noting the marked discrepancy between preclinical and clinical findings for mGluR2/3-targeting compounds: despite promising results in animal models, clinical trials in schizophrenia patients have largely been disappointing, with most agents showing little or no meaningful efficacy.

For example, re-treatment with LY354740 in healthy individuals receiving ketamine infusion was able to reverse ketamine-induced working memory impairments [238]. Pomaglumetad methionil (LY2140023), the oral prodrug of LY404039, a group II mGluR agonist, was tested in a 4-week phase II clinical trial involving 196 schizophrenic patients and improved both positive and negative symptoms compared to placebo [185]. When treatment duration was extended to 24 weeks [239], pomaglumetad methionil demonstrated similar efficacy to the current standard of care (including olanzapine, risperidone, or aripiprazole) in the first 8 weeks, although its effectiveness declined between weeks 16 and 24. Moreover, in a larger follow-up phase II trial with 1013 patients, this drug did not produce significant improvements over placebo, suggesting a partial loss of response over time, potentially reflecting adaptive changes in mGluR2/3 signaling [240]. A recent systematic review and meta-analysis, including four randomized clinical trials, confirmed that pomaglumetad methionil did not produce a statistically significant effect on Positive and Negative Symptoms Scale (PANSS) score compared to placebo (*p*-value  =  0.31), and was less effective than atypical antipsychotics (*p*-value  <  0.00001) [241]. These findings indicate that, despite its favorable profile with respect to weight gain, motor side effects, and prolactin elevation [242], this drug does not demonstrate consistent efficacy in the treatment of schizophrenia.

Regarding mGluR2-PAMs, two compounds have been tested in schizophrenia: AZD8529 and JNJ40411813 [243]. The former failed to improve symptoms relative to placebo but was generally well tolerated, with most adverse events being mild or moderate [242]. JNJ40411813 was tested in healthy volunteers treated with a low dose of ketamine, and was found to rescue ketamine-induced perceptual and behavioral changes [244]; however, when tested in patients affected by schizophrenia, JNJ40411813 met the primary safety and tolerability endpoints but resulted in being effective only in a small subgroup of subjects experiencing residual negative symptoms [226,245].

Finally, no clinical evidence is currently available for group III mGluR modulators in schizophrenia, as the only tested molecule is PXT002331, a mGluR4 modulator that failed to meet its primary endpoint in patients with Parkinson’s disease as an adjunct to L-DOPA [246].

Overall, while these molecules were generally well tolerated in human studies, their clinical efficacy of these mGluR-targeting strategies in schizophrenia remains limited. Further studies are needed to optimize dosing, patient stratification, and long-term effects before these agents can be considered viable treatment options.

## 6. Neurostimulation and mGluR Type I—PSD Protein Modulation

Brain stimulation has been proven to be efficacious for certain schizophrenia symptoms and cooperates synergistically with canonical pharmacological treatment in a clinical experimental setting [247]. Moreover, several reports indicate the effects on brain regions relevant for psychosis-like symptoms in a preclinical setting.

Multiple lines of evidence point to the possibility that different techniques of neuromodulation may significantly change the expression of mGluRs and PSD proteins. Transcranial direct current stimulation (tDCS) modulates cortical excitability, and a synergistic interaction between tDCS and pharmacologic mGluR5 facilitation has been reported. This mechanism may contribute, at least in part, to the chain of molecular events underlying these therapeutic effects.

Cathodal direct current stimulation (DCS) in vitro induces LTD (DCS-LTD) at excitatory synapses in both human and mouse neocortex slices. mGluR5 NAM abolish this effect, whereas mGluR5 itself contributes to and converts transient synaptic depression following DCS application into sustained DCS-LTD [248]. Further studies are needed to determine whether this effect is associated with changes in the expression of PSD proteins that interact directly with mGluR5, such as Homer1, or indirectly, as in the case of Shank and PSD-95. In contrast, vagal nerve stimulation has been shown, after sub-chronic treatment in rats, to induce changes in PSD that are not directly linked to mGluR receptors [249].

Transcranial magnetic stimulation (TMS) is an approved methodology for major depression by regulatory agencies of a few countries, and emerging research suggests a potential efficacy for negative symptoms of schizophrenia [250]. It has been shown that 10 Hz high-frequency repetitive TMS (HF-rTMS) ameliorated depression by inducing the expression of Homer1a and reducing the excitability of cortical pyramidal cells in an animal model of depression [251]. Chronic rTMS treatment during the 4 weeks significantly reduced the depression-like behavior. In slices obtained from the rTMS mice, normal excitability and Big K^+^ channel activity were recovered. It is is noteworthy to underline that GWAS and SNP analysis have associated Homer1 with cognitive symptomatology in patients with major depression [252]. Moreover, Homer1 knockout abolishes the efficacy of antidepressants in an animal model of major depression based on Adenosine1 receptor knockout [253]. Finally, antidepressants have been demonstrated to have moderate efficacy when added to antipsychotics to treat negative symptoms of schizophrenia [254].

The neuromodulation evidence shows significant heterogeneity across techniques, duration, species, disease models (schizophrenia vs. depression), and targeted symptom domains, with no unified mGluR/PSD mechanism established. This methodological diversity precludes definitive causal claims linking brain stimulation to consistent mGluR-PSD alterations in schizophrenia symptom relief.

## 7. Limitations, Conflicting Findings, and Open Questions

Although converging lines of evidence support a role for mGluR signaling and PSD-associated proteins in schizophrenia, several methodological and conceptual limitations temper current interpretations.

Post-mortem studies provide valuable molecular insights but are inherently limited by multiple confounding factors, including chronic antipsychotic exposure, duration of illness, substance use, metabolic comorbidities, and agonal state. These variables may independently influence mGluR expression, PSD protein levels, and synaptic architecture, complicating causal inferences. Moreover, most studies rely on small sample sizes and heterogeneous patient cohorts, reducing reproducibility.

Another major limitation of the available literature lies in the reliance on pharmacological NMDAR antagonism animal models (e.g., PCP, ketamine, MK-801), which capture selected endophenotypes (sensorimotor gating deficits, hyperlocomotion, working memory impairment) but do not recapitulate the developmental, cognitive, and negative symptom dimensions of schizophrenia. Consequently, compounds that normalize behavior in rodents may fail clinically because they target acute glutamatergic dysregulation rather than the complex synaptic and circuit-level alterations underlying chronic illness. This translational gap is exemplified by the discrepancy between robust preclinical effects of mGluR2/3 agonists and the limited efficacy observed in clinical trials. PSD proteins represent attractive conceptual targets because they integrate multiple receptor systems and regulate synaptic plasticity. However, pharmacologically modulating scaffolding proteins poses substantial challenges. These proteins are involved in numerous protein–protein interactions, and participate in essential physiological processes across brain regions. Intervening at this level risks widespread off-target effects; current compounds thought to “target” PSD mechanisms (e.g., SPG302) likely act indirectly by influencing spine dynamics or protein expression, rather than selectively modulating defined scaffold interactions.

A number of key mechanistic questions remain unresolved. First, it is still unclear whether alterations in mGluR signaling and PSD organization represent a primary pathogenic events in schizophrenia or rather secondary consequences of broader synaptic dysconnectivity. In other words, mGluR–PSD abnormalities could act as upstream drivers of circuit dysfunction, but they might also emerge as compensatory or maladaptive responses to ongoing network instability. A second open issue concerns the regional specificity of these alterations. Schizophrenia is characterized by distributed circuit abnormalities, yet different brain regions contribute to distinct symptom domains. Understanding how region-specific mGluR–PSD disruptions map onto clinical phenotypes remains a major challenge. Finally, it is not known whether mGluR-related abnormalities represent stable trait markers of vulnerability or are instead dynamically modulated across illness stages. Synaptic signaling pathways are highly plastic and may vary during prodromal phases, acute psychotic episodes, chronic illness, and in response to treatment. Clarifying the temporal dynamics of mGluR dysfunction could have important implications for identifying critical windows for intervention.

## 8. Discussion

Over the last three decades, the extended PSD hub, including type I mGluRs and their intracellular molecular partners, has gained attention in schizophrenia research for both pathophysiology and treatment development, as supported by different levels of experimental evidence ranging from post-mortem transcriptomic and proteomic to genomics and animal models of psychosis-like behavior. Importantly, schizophrenia should not be considered a biologically uniform disorder. Marked heterogeneity exists across individuals in terms of symptom profiles, illness stage, treatment response, and underlying neurobiological alterations. This variability complicates the interpretation of mGluR–PSD-related findings, as molecular mechanisms identified in post-mortem tissue, animal models, or small clinical cohorts may not generalize across the full spectrum of the disorder. It is therefore plausible that alterations in mGluR signaling and PSD organization are more relevant to specific subgroups—such as patients with treatment-resistant schizophrenia, prominent cognitive/disorganization features, or distinct developmental trajectories—rather than representing universal disease mechanisms.

This review was structured around four key questions aimed at clarifying whether mGluR–PSD protein complexes constitute a meaningful pathogenic and therapeutic hub in schizophrenia. Below, we summarize how the available evidence addresses each of these points.

(1) Even if it remains difficult to unveil the specific locus of mGluRs alteration in schizophrenia, few results emerge and represent a relevant starting point for further exploration, and when considered together, the available molecular, genetic, and pharmacological evidence—despite the substantial heterogeneity across methodologies, species, and clinical populations—supports the view that schizophrenia is not merely a disorder of neurotransmitter imbalance, but a disorder of synaptic nano-architecture in which mGluR-linked postsynaptic scaffolds play a central organizing role.
(2) mGluRs do not signal in isolation; their functional impact depends on their integration into multiprotein PSD assemblies. Constitutive Homer isoforms, Shank, GKAP, PSD-95, Norbin, PICK1, and Tamalin form a molecular lattice that anchors group I mGluRs in perisynaptic nanodomains, links them to NMDARs and Ca^2+^ stores, and coordinates receptor trafficking, endocytosis, and downstream signaling. This scaffolded organization allows mGluRs to regulate AMPAR trafficking, local protein synthesis, and Ca^2+^ microdomains, thereby shaping both LTP, LTD, and metaplasticity. Thus, this molecular mesh fine-tunes synaptic plasticity and metaplasticity, critical for maintaining dendritic spine integrity and arborization [74]. Disruption of this plastic network due to genetic variants, stress, or pharmacological perturbation altering receptor mobility, or uncoupling mGluRs from NMDARs may destabilize dendritic spines.
(3) The concept of multiscale connectivity, based on the organization of the CNS across multiple levels and layers, is an emerging framework that is still at an early stage of conceptualization. With the necessary caution, several lines of evidence may suggest that changes at the synaptic microdomain level, particularly at the PSD level, may propagate to large-scale connectivity changes [74,106,255,256]. When these microdomains are destabilized, synapses fail to appropriately tune their plasticity thresholds, leading to aberrant LTP/LTD and impaired metaplasticity. Over time, this may result in altered dendritic spine density and morphology, disrupted cortical microcircuits, and ultimately the functional dysconnectivity observed in neuroimaging studies of schizophrenia. In this model, large-scale abnormalities in prefrontal–striatal–thalamic networks may arise from cumulative failures of nanoscale synaptic organization.
(4) Although antipsychotics primarily target dopaminergic receptors, increasing evidence indicates that their molecular therapeutic effects involve direct or indirect modulation of mGluR–PSD protein complexes [4,10,74,257]. Clozapine, in particular, appears to exert broad effects on mGluR2/3, mGluR5, and glial mGluRs through epigenetic, metabolic, and immune-mediated mechanisms, which may contribute to its unique efficacy in TRS. Experimental interventions—such as mGluR1/5 positive allosteric modulators, aptamers, benzothiazole derivatives, and add-on compounds like D-cycloserine, D-aspartate, or minocycline—show distinct PSD gene modulation, representing promising avenues for adjunctive therapy [108,258,259]. Moreover, emerging compounds such as SPG302 further support this paradigm by directly enhancing PSD protein expression and spine stabilization, thereby promoting the reconstruction of glutamatergic microcircuits. These findings suggest that effective antipsychotic action requires not only modulation of neurotransmitter tone but also restoration of synaptic protein networks that govern plasticity and stability. Targeting the structural and functional integrity of these complexes may therefore open new avenues for biologically informed, circuit-oriented interventions, particularly in treatment-resistant forms of schizophrenia.

## 9. Conclusions

Current evidence on mGluR- and PSD-related mechanisms in schizophrenia spans different levels of translational certainty. Well-supported findings include the structural and functional coupling of group I mGluRs PSD components, the role of mGluR5 in regulating NMDAR function and AMPAR trafficking, and the modulation of PSD protein expression by antipsychotic treatment in preclinical models. Dysregulation of mGluR1/5 and their associated synaptic partners may affect micro-connectivity at dendritic spines, and could contribute to broader alterations in brain network connectivity that are relevant to schizophrenia pathophysiology. More tentative evidence suggests that restoring mGluR–PSD interactions could enhance synaptic stability, and that group II mGluR modulation might normalize cortical hyperglutamatergia; these hypotheses remain biologically plausible but should be validated in clinical populations. At a more speculative level, emerging therapeutic concepts include the direct pharmacological targeting of PSD scaffolding complexes, and the precision modulation of metaplasticity to promote symptom remission. However, further preclinical and clinical studies are needed to clarify the specificity and translational relevance of these approaches.

## Data Availability

No new data were created or analyzed in this study.

## Abbreviations

The following abbreviations are used in this manuscript:

WHO	World Health Organization
NMDAR	N-methyl-D-aspartate receptor
PSD	postsynaptic density
GABA	γ-Aminobutyric acid
mGluRs	metabotropic glutamate receptors
PDZ domains	PSD-95/Disks large/Zonula occludens-1 domains
TRS	treatment-resistant schizophrenia
PANSS	Positive and Negative Symptoms Scale
IP3	inositol trisphosphate (IP3)
DAG	diacylglycerol
cAMP	cyclic adenosine monophosphate
AMPARs	α-amino-3-hydroxy-5-methyl-4-isoxazole propionate receptors
GPCRs	G protein-coupled receptors
GTP	guanosine 5′-triphosphate
GDP	guanosine 5′-diphosphate
VFD	Venus Flytrap domain
LTP	long-term potentiation
LTD	long-term depression
EVH1	enabled/vasodilator-stimulated phosphoprotein homology
MAP	mitogen-activated protein
PIP2	phosphatidylinositol 4,5-bisphosphate
RyR	ryanodine receptor
BAR domain	Bin/Amphisin/Rys domain
IP3R	inositol triphosphate receptor
PSD-95	postsynaptic density protein 95
COS cells	CV-1 in Origin with SV40 cells
GKAP	guanilate-kinase associated protein
PLC	phospholipase C
PKC	protein kinase C
CICR	Ca^2+^-induced Ca^2+^ release
D2R	dopamine D2 receptor
D1R	dopamine D1 receptor
PKA	protein kinase A
mTORC	mammalian target of rapamycin complex
AP5	2-Amino-5-phosphonopentanoic acid
NO	nitric oxide
PFC	prefrontal cortex
5-HT_2A_R	serotonin 5-hydroxytryptamine 2A receptor
SNPs	single-nucleotide polymorphisms
GRM3	glutamate metabotropic receptor 3 gene
EAAT2	excitatory amino acid transporter 2
MRS	magnetic resonance spectroscopy
Glx	glutamate + glutamine
PCP	phencyclidine
NMRI mice	Naval Medical Research Institute mice
IκBα	Inhibitor of kappa B alpha
ERK1/2	extracellular signal-regulated kinases ½
Nf-κB	Nuclear Factor kappa-light-chain-enhancer of activated B-cells
Hdac2	histone deacetylase 2
L-BAIBA	L-β-aminoisobutyric acid
CA	Cornu Ammonis
BBB	blood–brain barrier
TMD	heptahelical transmembrane domain
CNS	central nervous system
CDPPB	3-Cyano-N-(1,3-diphenyl-1H-pyrazol-5-yl)benzamide
NAM	negative allosteric modulator
PAM	positive allosteric modulator
HPG	3-Hydroxyphenylglycine
CHPG	(R,S)-2-chloro-5-hydroxyphenylglycine
DFB	3,3-difluorobenzaldazine
MTEP	3-((2-Methyl-4-thiazolyl)ethynyl) pyridine
MPEP	2-methyl-6-phenylethynylpyridine
BINA	biphenylindanone A
GWAS	genome-wide association studies
PGC	psychiatric genomic consortium
tDCS	transcranial direct current stimulation
TMS	transcranial magnetic stimulation
DCS	direct current stimulation
